# Institute of Geology and Geophysics, Chinese Academy of Sciences—The time-space exploration from Earth core to galaxies

**DOI:** 10.1093/nsr/nwab030

**Published:** 2021-04-09

**Authors:** Wei Zhang, Weijie Zhao, Liang Zhao

The Institute of Geology and Geophysics, Chinese Academy of Sciences (IGGCAS) is located in the ancient city of Beijing, with the 700-year-old ancient city wall of the Yuan dynasty to the south and the prosperous Olympic Avenue to the east. Just as its location connects past and present, IGGCAS enjoys a long history as well as a brilliant future.

The current IGGCAS was established in 1999 by the merger of the Institute of Geology, CAS and the Institute of Geophysics, CAS, whose histories can be traced back to the Geological Survey of China established in 1913 and the Institute of Meteorology of Academia Sinica established in 1928, respectively.


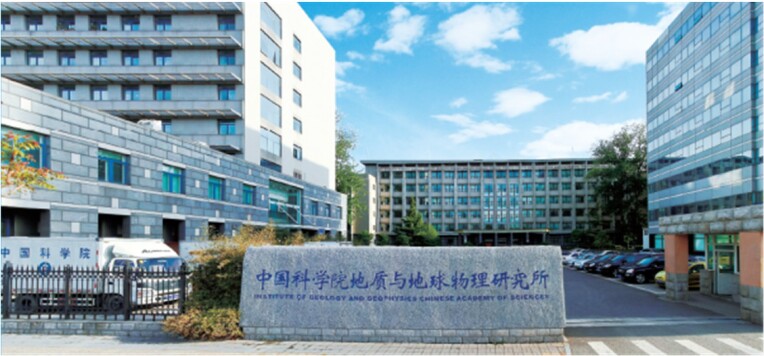


Throughout this century-long history, IGGCAS researchers made extraordinary contributions to the development of geoscience, the exploitation of resources and the construction of geological projects in China:

- IGGCAS pioneered the first research into geochemistry, seismology, geomagnetism, sedimentology, mineralogy, petrology and engineering geology in China.
- IGGCAS led the design of China's first satellite, contributed to the discovery of the Daqing Oil Field, the Shengli Oil Field and the world's largest rare-earth ore deposit, and also supported numerous major engineering projects such as the Three Gorges Dam, the Wuhan Yangtze River Bridge, the Daya Bay Nuclear Power Plant, the Chengdu-Kunming Railway, the Jinchuan Nickel Ore and the Comprehensive Harnessing Project of Huai River.
- Since the foundation of CAS, there have been 252 CAS academicians in geoscience, among which around 80 are working or have worked in IGGCAS.

After decades of rapid development, IGGCAS has become a modernized comprehensive research institution harboring seven research departments and more than 60 laboratories. It is strong in geology, geophysics, geochemistry, geological engineering and planetary science. It is equipped with comprehensive and advanced instruments capable of detecting deep earth, deep space, deep ocean and deep time. With strong funding support from the CAS, the National Natural Science Foundation of China (NSFC) and the Ministry of Science and Technology of China (MOST), IGGCAS researchers are leading cutting-edge research that explores the Earth's innermost core and extends right out to the fringes of galaxies.


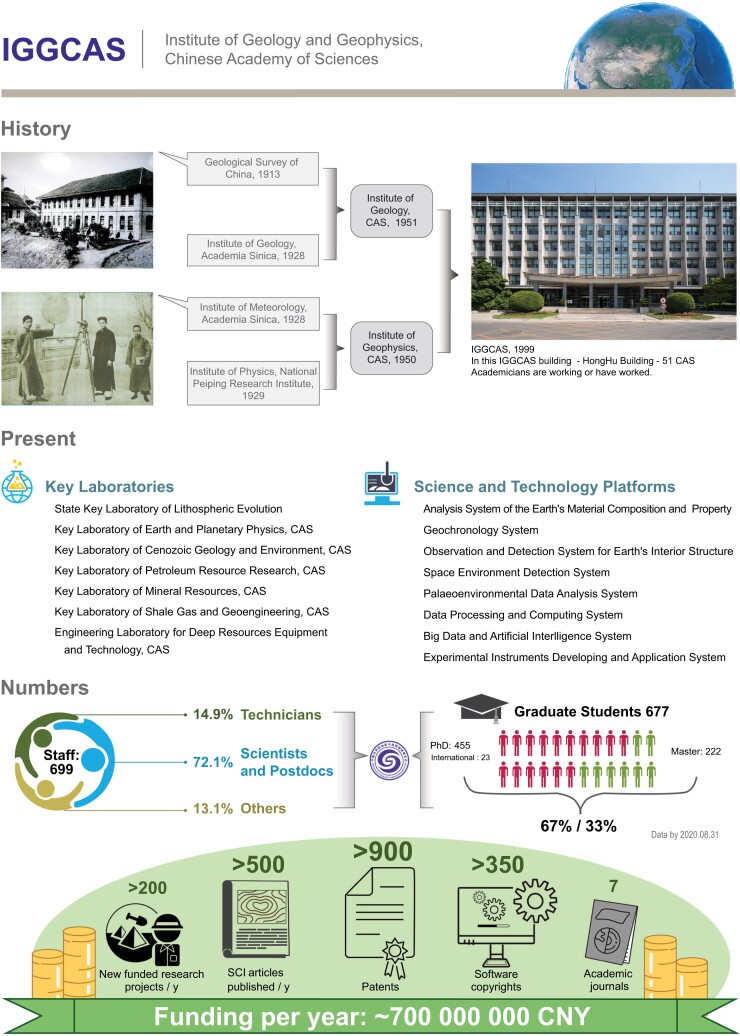


## RESEARCH IN IGGCAS: EARTH, LIFE AND SPACE

### Earth in time

IGGCAS scientists first introduced plate tectonics theories to the Chinese scientific community and used them to elaborate on the geological evolution of the Tibetan Plateau in the 1970s. Through its history, IGGCAS has been leading the development of solid earth sciences in China.

**Figure:**
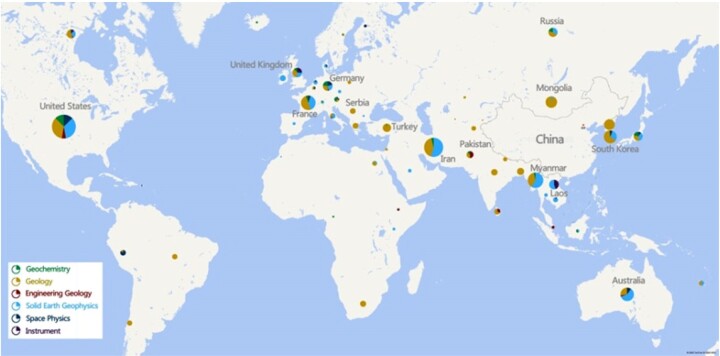
International Tethyan research throughout the Belt and Road (2011–20).

With the establishment of the State Key Laboratory of Lithospheric Evolution in 2005, IGGCAS has made a series of advancements on the mechanisms underlying plate movement and the formation of lithosphere. Especially, their studies on the destruction of the North China Craton and the geological evolution of Precambrian attracted international interest and became active research topics.

**Figure:**
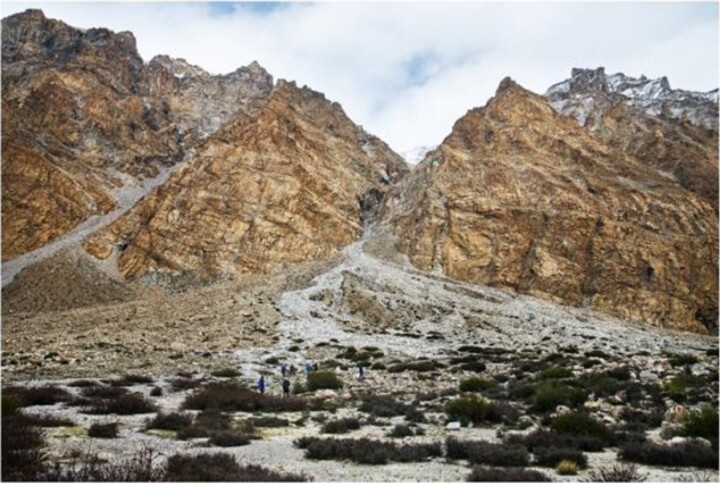
Geochemical study of the Himalayan Leucogranite (Photo/Hui Ren).

IGGCAS also led a series of international collaboration projects to explore the geology, geophysics and geochemistry of the Alps, the Iranian Plateau, the Tibetan Plateau, the India–Myanmar Range and northwest Australia along the ancient Tethyan belt. They also deployed self-developed submarine seismographs in the China offshore, the Indian Ocean, the deepest Mariana Trench, the Caspian Sea, etc. So far, the Tethyan tectonic domain running through multiple continents has become a new research frontier pioneered by IGGCAS.

**Figure:**
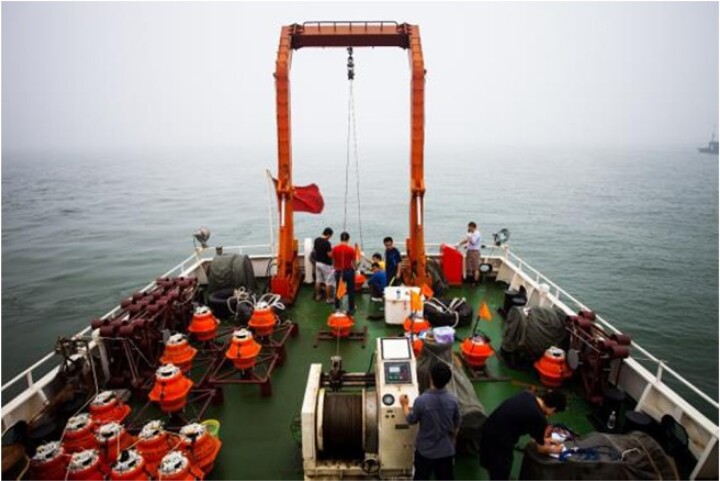
A self-developed submarine seismograph is being deployed (Photo/Tianyao Hao).

#### Highlight: the destruction of the North China Craton

The North China Craton has long attracted academic interest for its unique properties. This 1 500 000 km^2^ stretch of continental block has been unstable since an abrupt thinning and destruction in the Mesozoic era, a phenomenon known as ‘craton destruction’ or ‘decratonization’, a geological process essential in the Earth's evolution. IGGCAS researchers showed that the vigorous crust deformation and magmatism of the North China Craton can be associated with the thinning lithosphere. Its loss of stability has been considered as a possible cause for the 1976 Tangshan earthquake in the region. IGGCAS has also uncovered the link between this defining episode and the genesis of various mineral resources in the region.

**Figure:**
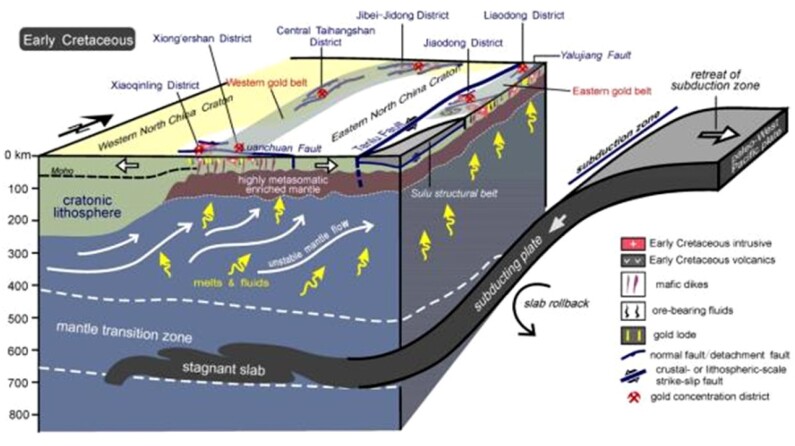
Deep tectonic processes of the North China Craton and relationship between decratonization and gold mineralization in the Early Cretaceous. Adapted with permission from [1].

#### Highlight: laser micro-isotope studies

Isotope geochemistry and geochronology are important tools for accurately determining the time and tracing the formation and evolution process of the Earth. The research team led by Prof. Jin-Hui Yang developed a series of high-precision laser micro-beam techniques including Sr-Nd-Hf isotopic analysis and simultaneous determinations of age, isotopes and trace elements. These techniques were used to reveal many complex geological processes such as petrogenesis of magmatic rocks and cratonic destruction, and provided new methods and insights for understanding complex geological processes from a microscopic perspective.

**Figure:**
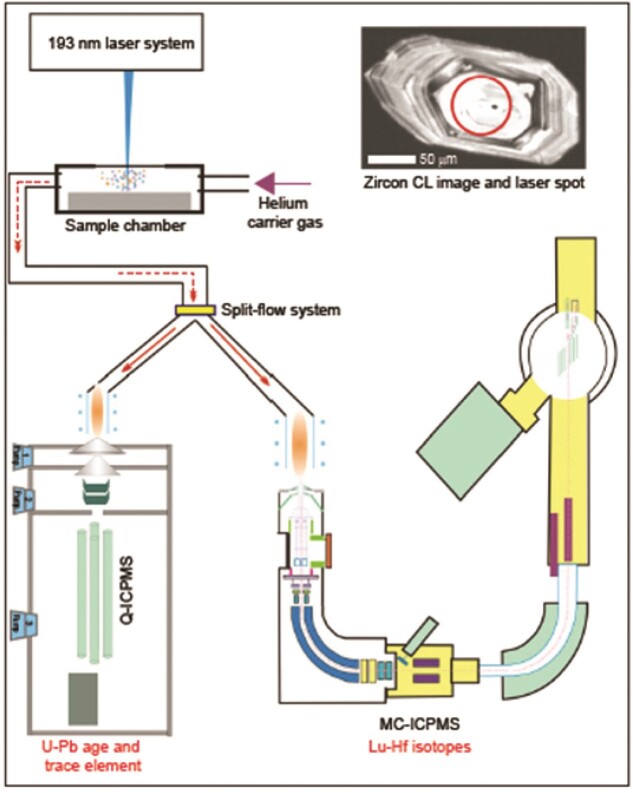
Simultaneous determinations of U-Pb age, Hf isotopes and trace element compositions of zircon by laser-ablation micro-beam technique.

### Understanding habitable environments

A habitable environment is what makes the Earth unique among planets, and a better understanding of ancient climate and ecological systems will improve our understanding of future climate change. Traditionally, we get such information from deep-sea sediments and ice cores.

**Figure:**
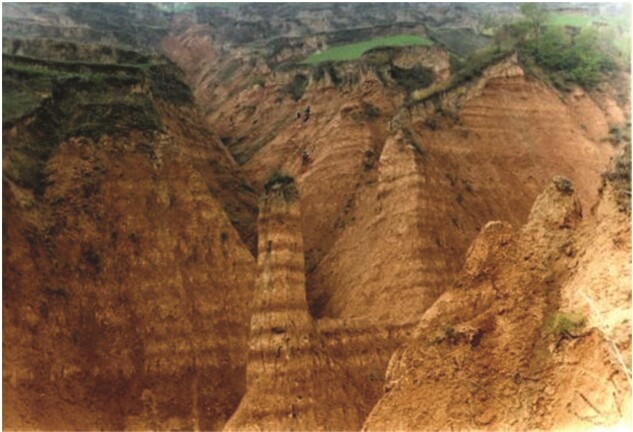
Miocene loess in Qin’an, Gansu Province (Photo/Shuzhen Peng).

However, based on the fundamental contributions by IGGCAS researchers led by Prof. Tungsheng Liu (1917–2008), loess, or sediment formed by wind-blown silt, has been added to the list of research resources.

**Figure:**
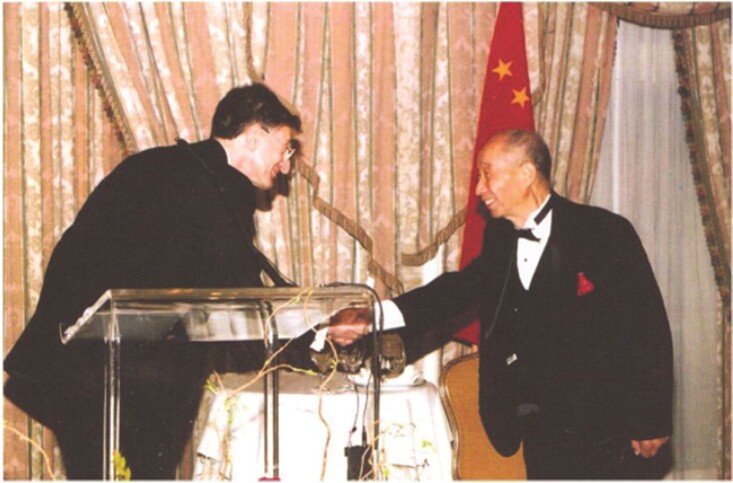
Prof. Tungsheng Liu won the Tyler Prize for Environmental Achievement in 2002. He is the first Asian scholar to win this prize.

Liu's team was also one of the teams that first connected the dots between environmental changes and human welfare, and established the field of environmental geology in China. His work promoted disease prevention and research on past climate changes. This work led him to win the Tyler Prize for Environmental Achievement in 2002 and the State Preeminent Science and Technology Award in 2003.

**Figure:**
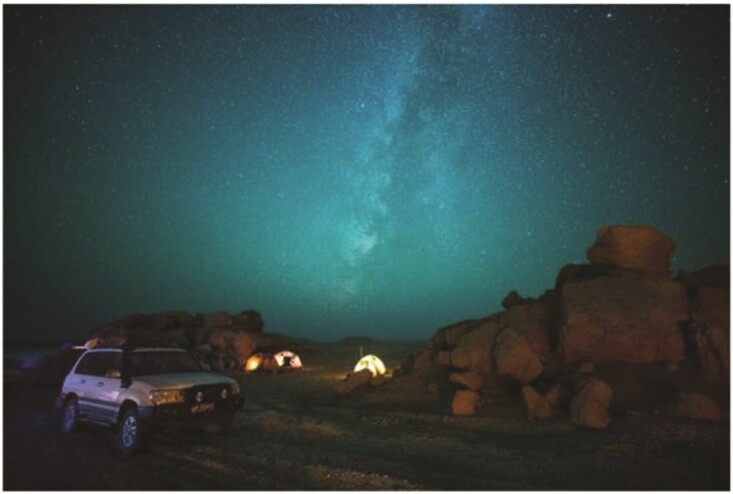
Environmental archaeology research in Lop Nor (Photo/Hui Ren).

IGGCAS researchers have contributed to the unlocking of mineral, petroleum and gas resources. They made substantial contributions to the discovery and exploration of the Pugang gas field in Sichuan, Jiaodong gold ore field in Shandong, Chalukou molybdenum deposit in Heilongjiang (the third-largest in the world), and Bayan Obo in Inner Mongolia (the world's largest rare-earth deposit). For national engineering projects, such as the construction of the Three Gorges Dam and the South-to-North Water Diversion Project, IGGCAS leads site evaluation and geohazard prevention. A theory of rock mass geomechanics was also founded during these engineering processes.

**Figure:**
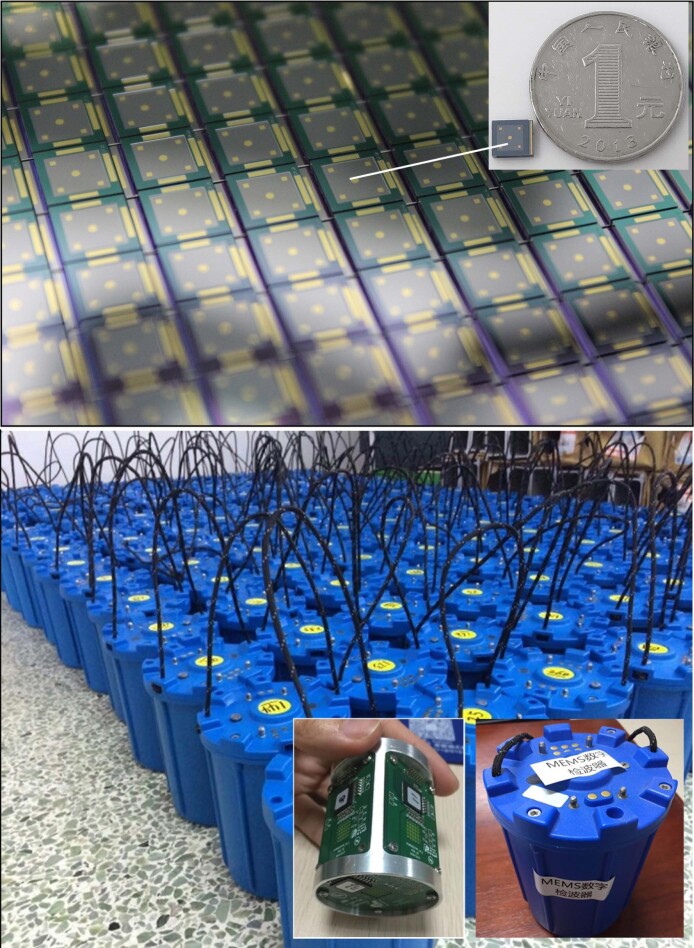
MEMS chip and geophone.

IGGCAS also developed novel technologies and equipment for deep-earth exploration, which will be helpful for understanding and improving our living environment.

**Figure:**
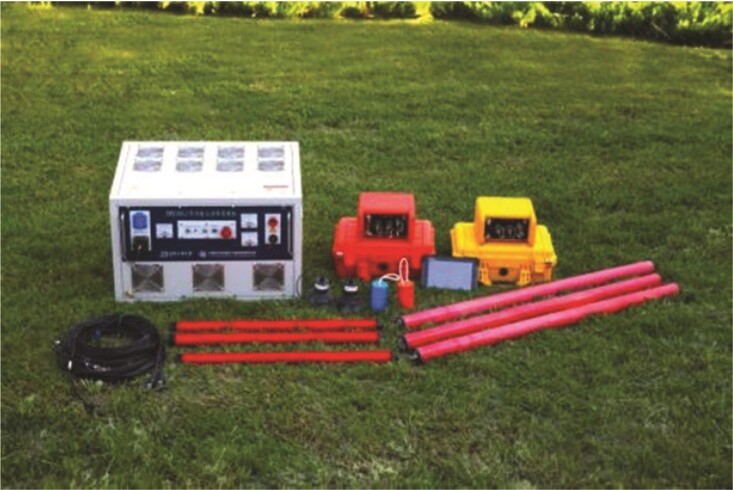
Surface electromagnetic prospecting system.

**Figure:**
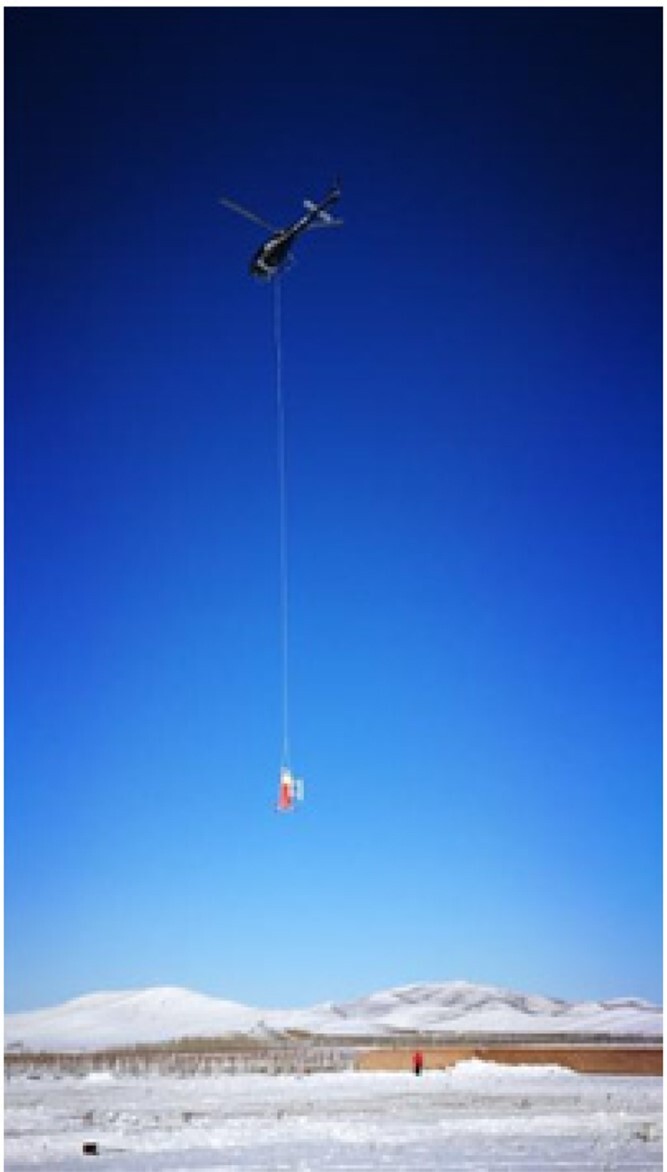
Superconducting aeromagnetic system

#### Highlight: East Asian monsoons and Asian interior deserts

The origins and evolutionary mechanisms of the monsoon zone in eastern and southern China and the desert zone in inland northwest China are of great scientific interest to the academic community. A team led by IGGCAS Prof. Zhengtang Guo studied Miocene aeolian sediments and traced the history of the monsoon environment and inland deserts in Asia from 8 million years ago to 22 million years ago. They also suggested that the uplift of the Qinghai-Tibet Plateau was the main trigger. This allowed insightful understanding of the onset and development of East Asian monsoons and Asian interior deserts. The reconstruction of the evolutionary history of both provides a paleoclimatological basis for the development trend and impact assessment of global change.


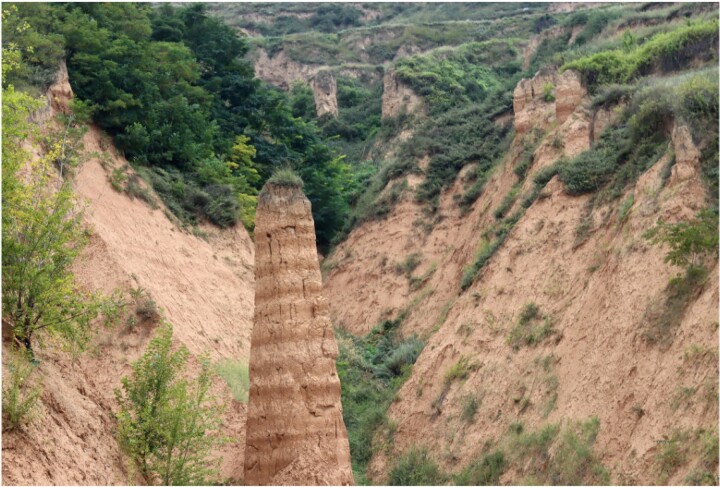


#### Highlight: seafloor geophysical instruments

Seafloor geophysical investigations can illuminate the deep-earth interior in the oceanic area. During decades of technical research led by Profs. Tianyao Hao and Qingyu You, a series of seafloor geophysical instruments have been successfully developed, including multiple types of Ocean Bottom Seismometer (OBS), Ocean Bottom Magnetometer (OBM), Ocean Bottom Electromagnetic Receiver (OBEM), etc. These seafloor instruments have been widely used in geophysical surveys and seafloor observation network experiments. In 2017, the 10 000-meter OBS was successfully applied in the survey of the Mariana Trench Challenger Abyss, and completed two 10 000-meter class active source seismic profiles which became a milestone of marine geophysics in China.


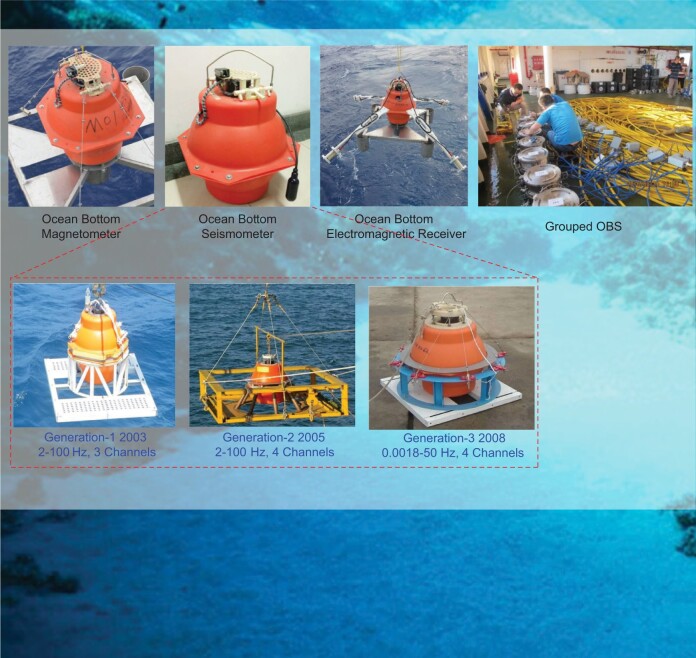


Series seafloor geophysical instruments (Photo/Qingyu You).

### From deep earth to deep space

IGGCAS has been constantly playing a pivotal role in China's space exploration projects, from the first artificial Earth satellite, to the ongoing Chinese Lunar Exploration Program, the recently launched Mars mission, and the coming asteroid sampling program.

Comprehensive observatories built by the IGGCAS are providing unprecedented data sources for investigations of geomagnetic field, ionosphere and atmosphere. The World Data System (WDC) for Geophysics, Beijing hosted by IGGCAS is a regular member of ISC-WDS. It is certified by CoreTrustSeal and recognized as an official repository by the American Geophysical Union (AGU). IGGCAS also houses the Center of Big Data and AI for Earth Science, allowing big data analysis on geoscience.

**Figure:**
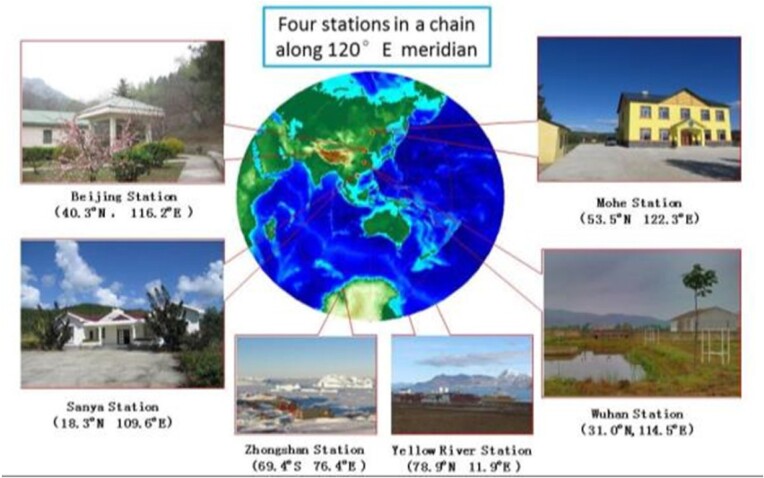
Space environment observatories.

China has formed a roadmap for planetary exploration. IGGCAS has prestigious research groups in space environments, planetary physics and planetary chemistry. As the leading institute in sciences for the Mars mission ‘Tianwen-1’, IGGCAS will undoubtedly have an extraordinary influence on planetary sciences and the understanding of planetary habitability.

**Figure:**
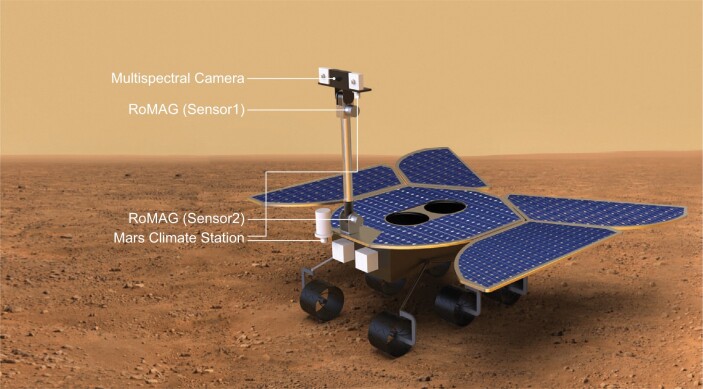
Mars magnetic field measurement (artistic illustration). Adapted with permission from [2].

#### Highlight: comparative study of planetary magnetic fields

Unlike other planets in our solar system, the Earth contains a large amount of liquid water. It has been suggested that the magnetic field of a planet can play an important role in preventing water from escaping, for example, the magnetic reconnection process may send the escaped charged particles back to the planet. A research team in IGGCAS, led by Prof. Yong Wei, conducted research using observations and numerical simulations to understand the widely divergent magnetic fields of different planets. Their results indicate that the location of magnetic reconnection, and the reconnection occurrence rate, can be very different for different planets. The Mars crust is more intensely magnetized than that of the Earth, thus may have strong influence on atmospheric escape. Prof. Aimin Du led the development of the magnetometer on board the rover of the Tianwen-1 mission, to measure the magnetic field on the surface of Mars for the first time.

**Figure:**
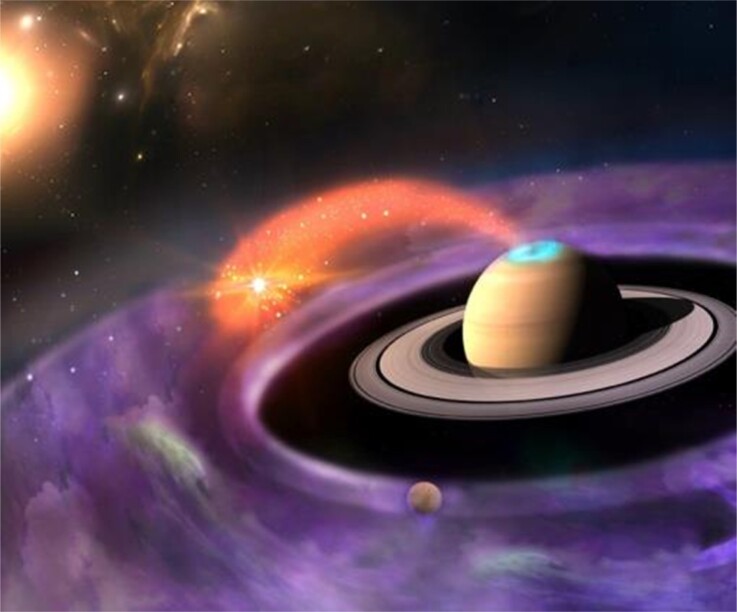
Dayside magnetodisc reconnection creates a routine for particle acceleration and transport between Saturn and the ambient space (artistic illustration). Adapted with permission from the cover image of *BCAS (Chinese Version)* 2019, Vol 34 No 7.

### Future directions

In 2015, IGGCAS proposed an ambitious plan to establish an international research center integrating the missions of theoretical innovation, technical development, public interest and geoscience education. IGGCAS will hold its traditional excellence in solid-earth research, and also place its focus on comprehensive scientific issues including deep earth, deep space, deep ocean, deep time, natural resources and sustainable development.

**Figure:**
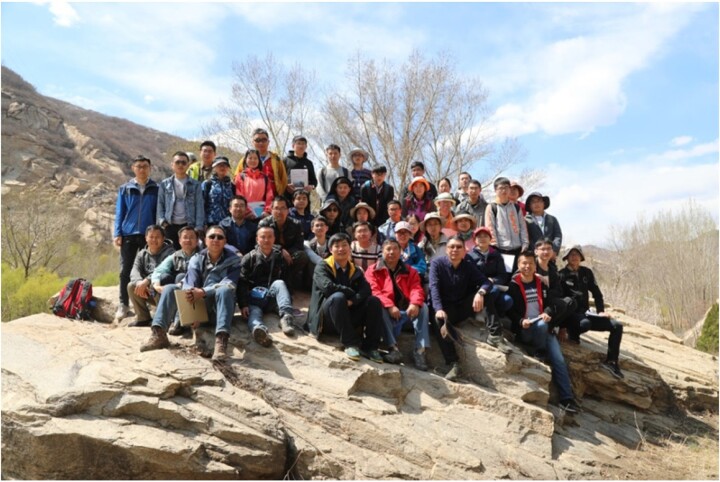
As a leading institute, IGGCAS has been combining science, technology, education and public communication to promote geoscience in China and beyond (Photo/Hui Ren and Tiesheng Li)

In order to fulfill this new visionary plan, IGGCAS has committed to the transformation of research concepts, organization structure, management models and innovation patterns. Taking a global perspective, IGGCAS is dedicated to five scientific goals:

1. Earth system science, such as Tethys tectonic evolution and its effect on energy and mineral resources;
2. Planetary science, especially lunar and Mars exploration;
3. Understanding habitable environments;
4. Fundamental research of critical resource and energy;
5. Advanced deep resource exploration technology and equipment development.

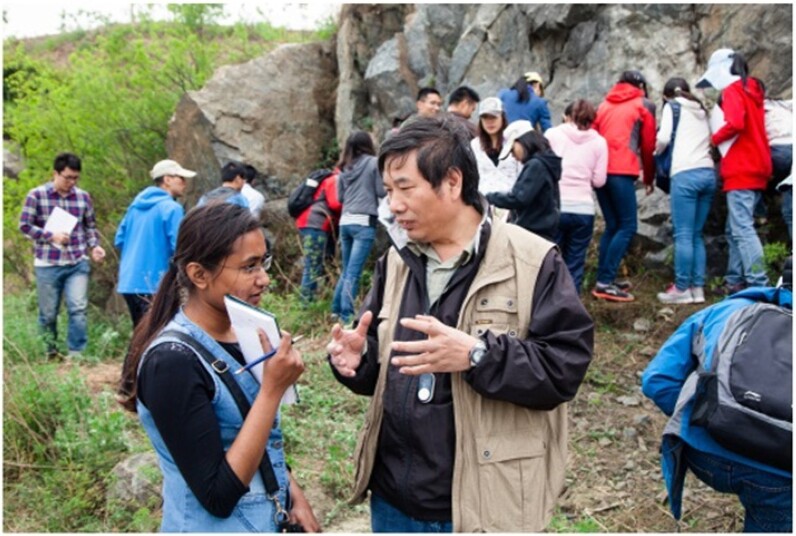


IGGCAS is willing to collaborate with geological scientists from all over the world and explore the very frontiers of Earth and planetary sciences.

## ENERGETIC RESEARCHERS IN THEIR 30S

**Shuhui Cai: Constraining the chronology of ancient civilizations**

Shuhui Cai, born in 1985, is an associate professor working on archeomagnetism. She loves archeological relics such as kilns and potteries because they preserve evolutionary information of the geomagnetic field. She has recovered a large amount of paleomagnetic data spanning the past millennia from multiple Chinese relics and established the first archeointensity reference curve covering the past 7000 years for Eastern Asia. The reference curve constitutes the foundation for understanding regional features of the geomagnetic field and archeological dating in this area. She is devoted to establishing centennial variations of the geomagnetic field during the Holocene in Eastern Asia, which can be used to constrain the chronology of ancient civilizations as well as to predict future variations of the geomagnetic field.

**Figure:**
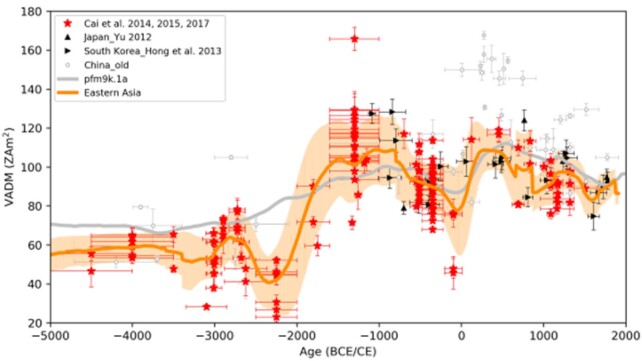
Reference curve of geomagnetic intensity during the past 7000 years in Eastern Asia (Picture/Shuhui Cai).


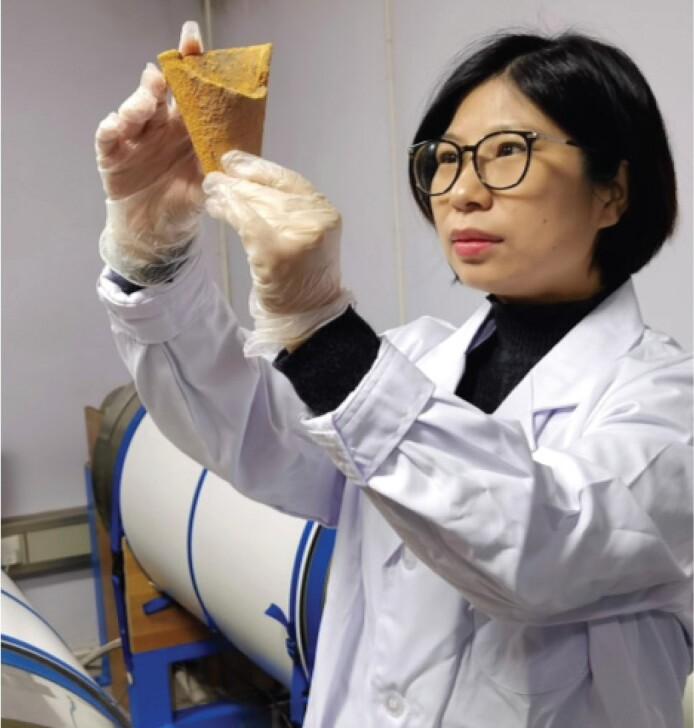


**Bo Wan: Why are plate tectonics global?**

Plate tectonics describe the motions of rigid plates on Earth's surface. However, it is debated what drives the plates to move and when the plate network became a global phenomenon. Dr. Bo Wan, born in 1982, is a Professor of geology in IGGCAS. He has assessed the geology and geophysics of the ancient Tethyan Realm, which involved the tectonic motions of plates from East Asia to West Europe. He proposed that subduction—the thrusting of one plate over another—has been the main driver of plate motion over the last 500 million years. He did not stop there. By discovering key evidence in even deeper time, he traced the global subduction network back as far as 2 billion years. He is now investigating how Earth evolved into its modern form of plate tectonics, which is unique among terrestrial planets.

**Figure:**
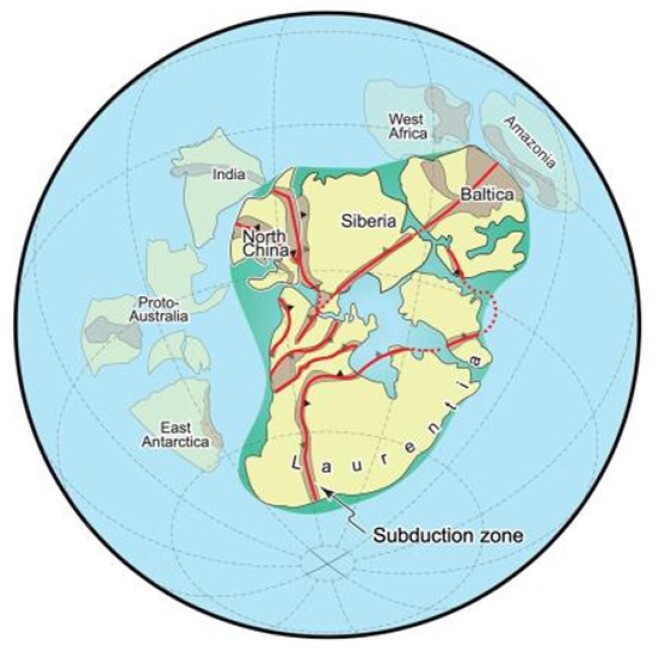
Global subduction-driven plate tectonic network at 1.8 Ga ago (Picture/Bo Wan).


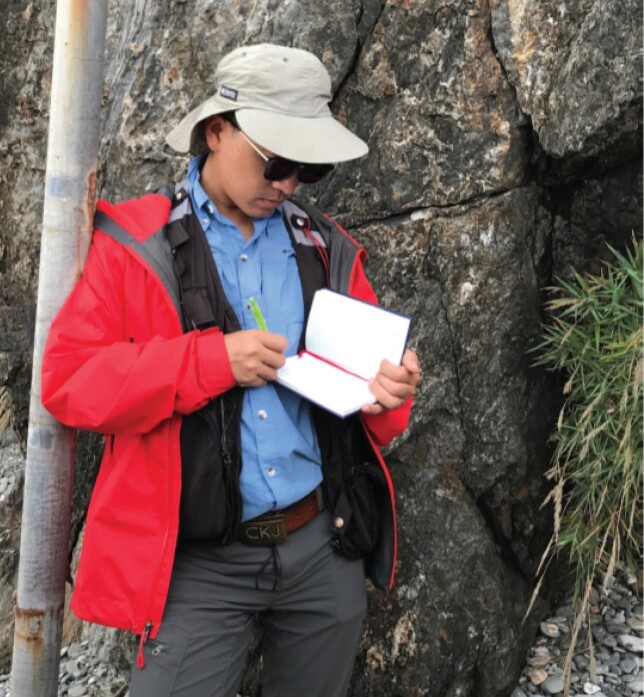


**Chenxi Xu: Climate changes written in tree rings**

Prof. Chenxi Xu, born in 1985, mainly focused on climate changes in the past millennium. He has been using oxygen isotope in trees in low latitude areas to investigate its potential to reconstruct past climate changes since he was a graduate student. His research reveals that tree ring oxygen isotope can be used for cross-dating trees in tropical Asia, which are difficult to cross-date using tree ring width and is a promising proxy of Asian summer monsoon. He has reconstructed the South Asia summer monsoon history of the last 400 years. He also works for international organizations such as PAGES (Past Global Changes) and INQUA (International Union for Quaternary Research). The aim of his current research is to reconstruct a history of global monsoons that affect almost 3 billion people worldwide and to provide insights on the mechanism of monsoon changes.

**Figure:**
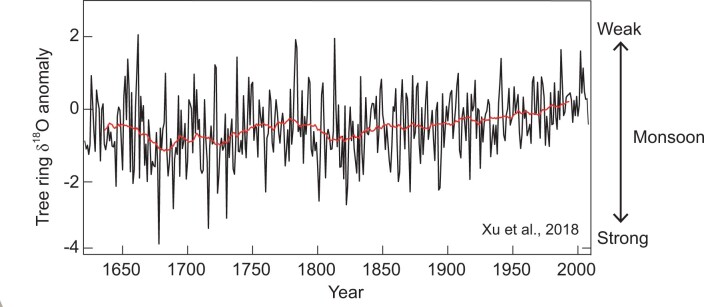
South Asia summer monsoon changes revealed from tree rings in the past 400 years (Picture/Chenxi Xu).


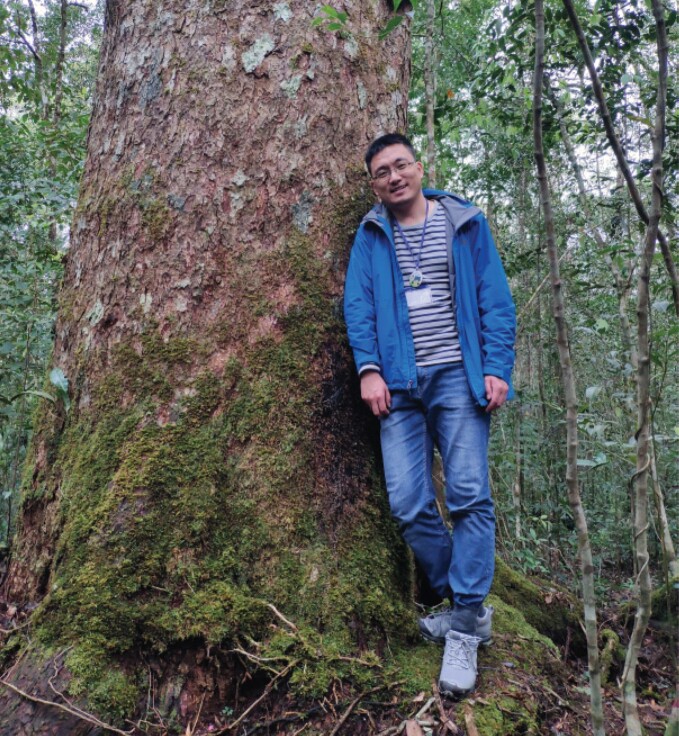


**Wei Lin: Microbial magnetoreception and life in extreme environments**

Wei Lin, born in 1983, is a professor of geobiology and astrobiology at IGGCAS Key Laboratory of Earth and Planetary Physics. His interests include the origin and evolution of microbial magnetoreception and life in extreme environments. He and his colleagues discovered a group of novel magnetotactic bacterial lineages from various ecosystems, expanding the genomic diversity of magnetosensitive microorganisms and deepening our understanding of the origin and evolution of magnetoreception. His current research interests focus on life in Mars-analogue environments on Earth, such as the Near Space and hyperarid environments.

**Figure:**
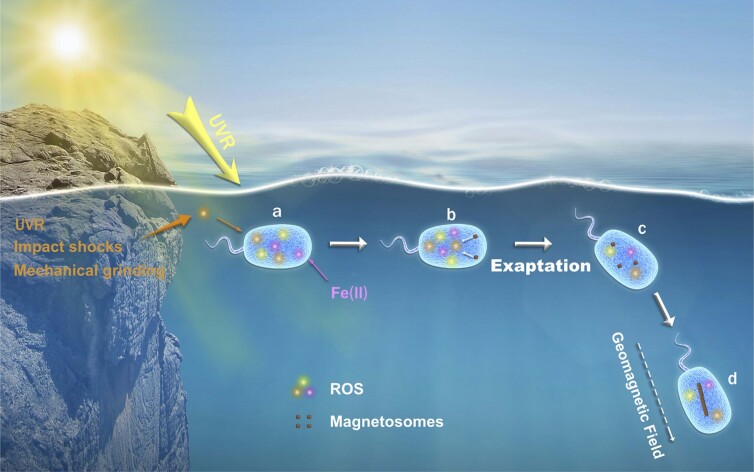
The origin and evolution of microbial magnetoreception. Adapted with permission from [3].


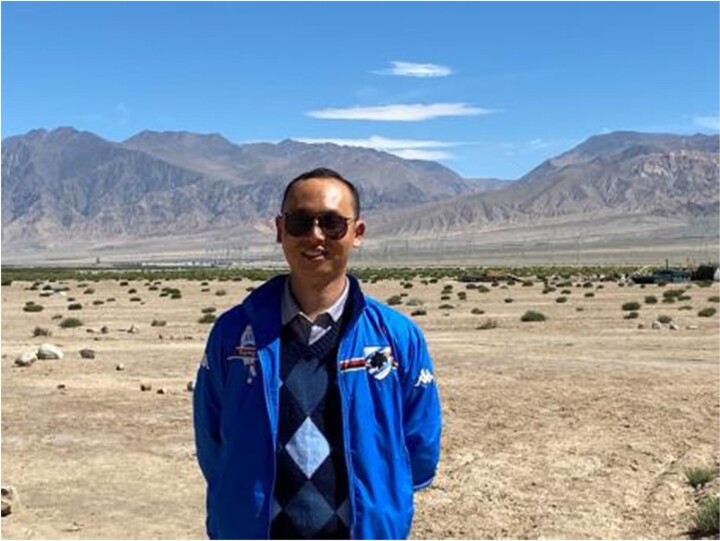


## INTERVIEWS

Fuyuan Wu (1962-), Director of IGGCAS, Professor of Geology, Academician of CAS (Photo/Hui Ren).

***IGGCAS is extending its research scope from China to the globe. Why is it important and how would the institute organize international cooperation?***


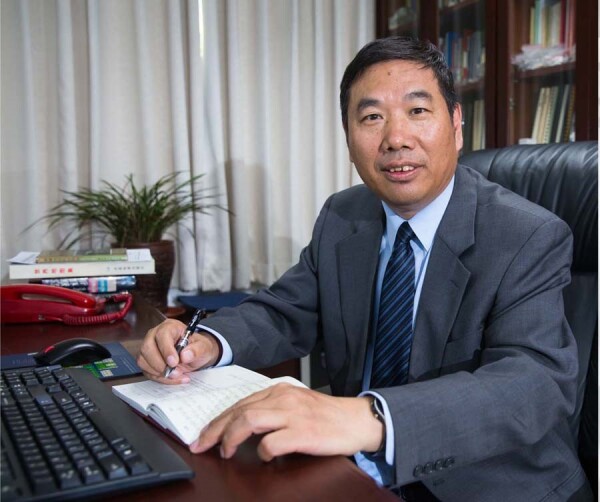


I will say there is no national boundary for earth science. The ideal natural laboratory of earth science would be a certain corner in the Earth or even in a planet, which depends on the scientific goals we are pursuing. That is the reason why the global view is so important for earth science. In the next decade, IGGCAS is going to lead several international projects including the Tethys dynamics, as well as exploring the moon and Mars. Some key projects sponsored by CAS, NSFC and MOST will provide unprecedented support for global geoscientists to work together on these frontier topics.

***What kind of research environment does IGGCAS aim to provide for its researchers?***

We are enormously proud of the research environment that our senior scientists have built and handed over to us, which is characterized as patriotic, initiative, inclusive, impartial and so on. With this environment, it is well known that IGGCAS is an ideal platform for young and senior scientists to realize their ambitions. And I think our generation will continue pursuing goals to build a progressive environment suitable for advanced research supporting national needs and frontier sciences.

***Geology is an old branch of science. How would it develop in the future?***

Speaking of history, geoscience is an old branch of science, but it is still full of energy and frontier ideas. The development of our country and society will rely on the breakthroughs of earth science more and more. In the future, geoscience will evolve into a systematic science that formulates the dynamical evolution of multiple spheres within and outside of the Earth and planets. It will provide more fundamental support for national needs and service public interests.

Patrick Rioual (1971-), Professor of Quarternary Geology.

***Is China a good place to study diatoms and environmental change on our planet?***


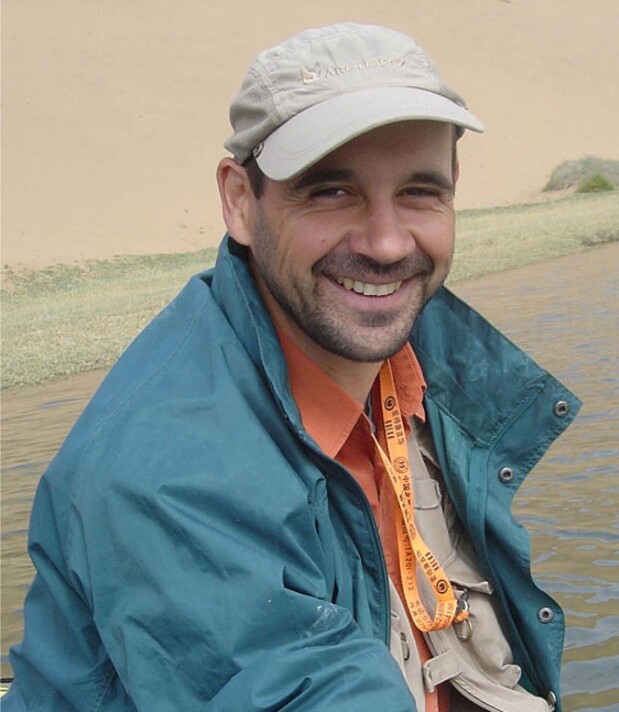


My research focuses on diatoms, which are microscopic unicellular algae that can be found in all aquatic environments (oceans, lakes, rivers, even on wet soils). I use the remains of these organisms that are buried in lake sediments as indicators for past changes in environment and climate. China has many different types of environments and climates. This leads to a great diversity of lakes such as volcanic and glacial lakes, or even groundwater-fed lakes in deserts, and to an even greater diversity of diatoms. Many diatom species remain to be ‘discovered’ and scientifically described. Their ecology and evolution also need to be investigated for interpreting the past changes we observed in the sediment sequences. For that reason, it is very interesting and challenging to work on diatoms in China.


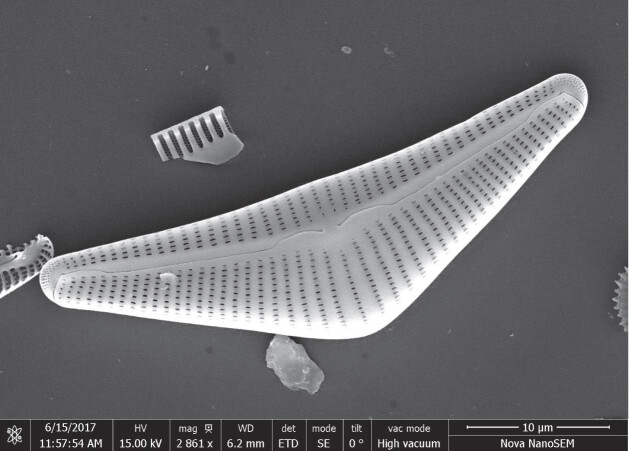


***How does IGGCAS support your research and life in China?***

Diatom research is more generally associated with the fields of biology and ecology, so obviously it is not a core subject for the IGGCAS but I can still benefit from the excellent facilities available here. In particular, I have access to several high-performance scanning electron microscopes. This equipment is necessary to investigate diatoms. Being part of IGGCAS also allows me to take part in several large, well-funded research projects in collaboration with my colleagues who are geochemists, geomorphologists and paleoclimatologists.

***What is the biggest difference between IGGCAS and European research institutes?***

Nowadays, the methods for sampling and analyzing are often very standardized to meet the criteria for scientific publications. Therefore the way to do science is very similar in all the laboratories around the world. For me, the biggest difference would be that I do not have to teach and that I can concentrate all my efforts on research. This situation is quite uncommon in European academic institutes.

Dongmei Tang (1981-), Associate Professor of Geology.

***What is your research interest and why is it important?***


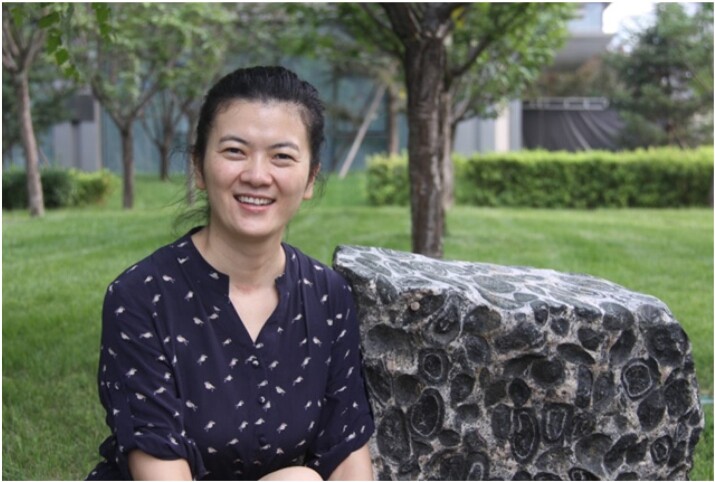


My interest is magmatic nickel-copper-(PGE) sulfide deposit. Nickel is one of the strategic metals, and platinum group elements are critical metals. Ni and PGE have immeasurable values for China's development and power of discourse in the world. Research of nickel-copper-(PGE) deposits in our country is of great significance in establishing metallogenic theory and deepening our understanding of the origin of this kind of deposit, thus effectively guiding mineral exploration.

***How could the research of IGGCAS scientists help with the mineral resources industry? Any examples?***

Scientists of IGGCAS insist on studying the deposits of economic values, guiding mineral exploration of the mineral resources industry and promoting deep metal prospecting, developing and improving our metallogenic theory according to the results of industrial exploration. For example, the Bayan Obo giant rare-earth element (REE) deposit is the largest REE deposit in the world. Three new minerals have been discovered and named after the discoverers, who are pioneering economic geologists of our institute. In recent years, our institute has set up a joint research center for strategic critical metals study. The results will be directly shared with the mineral resource industry for further ore prospecting and recycling.

***You have been studying and working in IGGCAS since 2006. What are the major developments of the institute throughout these years?***

The North China Craton destruction, space physics and Quaternary subjects lead the international and domestic frontier. The resources of our institute have been largely integrated, aiming to solve major and basic scientific problems of deep earth and remote space. CAS academicians take a leading role and high-end talents bring fresh expertise. Undertaking important national projects of MOST, CAS and NSFC makes the institute more famous and recognized. Plenty of scientific achievements such as national awards are achieved. The world-class equipment has been established and improved during these years.

Zhonghua Yao (1986-), Special-Term Professor of Planetary Sciences.

***Why did you join IGGCAS? What are the characteristics of the institute that attracted you?***


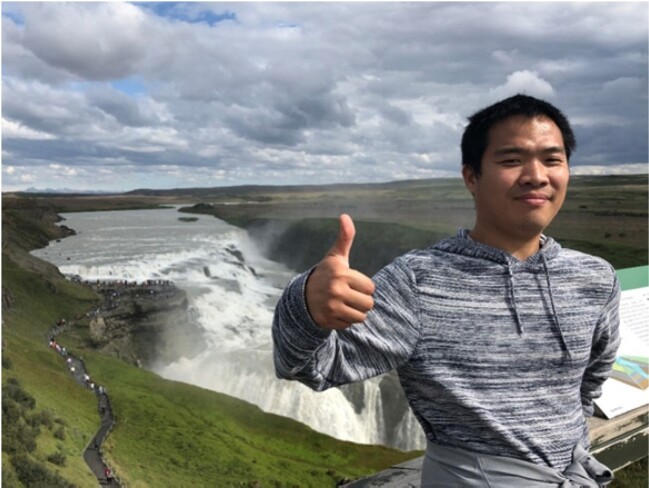


Our country always has a deep space dream, and now it seems that the dream is being achieved. I cannot miss such an opportunity of a lifetime, so I decided to terminate my trip abroad in 2019. My major is planetary science, and I particularly wish to make my own contributions in planning scientific goals for our national space exploration missions. IGGCAS is one of the leading institutes in planetary exploration, and has many chief scientists. It was not a tough decision to join IGGCAS. I knew that I would enjoy my career after joining IGGCAS.

***How does IGGCAS help young scientists to start their own labs?***

Nowadays, young scientists are suffering high pressure all over the world. Many talented colleagues in Europe and the US could not find a faculty position because of the shrinking research budgets in our field. Young researchers are struggling for their next contract, and it is more and more difficult to make a long-term plan for their careers. In IGGCAS, there are many senior researchers planning for national strategies, allowing young scientists to easily connect their research with national needs and allowing young researchers to learn how to plan for high-level projects.

***What is your current research interest?***

During my research experience, I have always been thinking of three things: (i) what sciences are important to the country and to human beings, (ii) what is my most enjoyed research, (iii) which topics could influence most of the community. I would like to describe my research interest as an evolving research interest. I currently work on giant planetary space environments, and their coupling processes with geological activities from planetary moons, i.e. Jupiter's moon Io and Saturn's moon Enceladus. With the strong support from IGGCAS, my collaborators and I are building telescopes for observing planetary environments in Qinghai.

Art editor: Xiaoling Yu (NSR).

## Author Biographical

Wei Zhang (IGGCAS), Weijie Zhao (NSR) and Liang Zhao (IGGCAS)

## References

[1] Zhu RX , Fan HR, Li JW et al. Sci China Earth Sci 2015; 58: 1523–37.

[2] Du AM , Zhang Y, Li HY et al. Space Sci Rev 2020; 216: 135.

[3] Lin W , Kirschvink JL, Paterson GA et al. Natl Sci Rev 2020; 7: 472–9. 10.1093/nsr/nwz065 PMC8288953 34692062

